# The relationship between hemogram based inflammatory indices and prognosis in ectopic pregnancy cases treated with methotrexate

**DOI:** 10.1038/s41598-025-09149-x

**Published:** 2025-07-02

**Authors:** Ceren Bilir, Cenk Soysal, İsmail Bıyık, Özlem Ulaş, Nuh Mehmet Erbakırcı, Hatice Sarı, Yasemin Taşçı

**Affiliations:** Department of Obstetrics and Gynecology, Kutahya Health Sciences University, Kütahya Sağlık Bilimleri Üniversitesi Tıp Fakültesi Dekanlığı Evliya Çelebi Yerleşkesi Tavşanlı Yolu 10. km, Kutahya, Turkey

**Keywords:** Ectopic pregnancy, Methotrexate treatment, Systemic immune-inflammation index, Inflammatory indices, Rupture, Reproductive disorders, Inflammatory diseases

## Abstract

This study aimed to evaluate the effectiveness of β-hCG levels and inflammatory indices (NLR, MLR, PLR, and SII) in predicting rupture risk and treatment success in ectopic pregnancies treated with methotrexate (MTX). A retrospective analysis was conducted between 2017 and 2024 at Kütahya Health Sciences University, including 85 patients diagnosed with ectopic pregnancy. The diagnosis was confirmed by transvaginal ultrasonography. Patients with non-tubal ectopic pregnancies, active infections, autoimmune diseases, or organ failure were excluded. Demographic data, β-hCG levels (at admission, treatment day, and days 4 and 7), and inflammatory parameters were recorded. ROC analysis was used to assess rupture risk, with significance set at *p* < 0.05. The mean age was 32.1 ± 6.3 years, and the mean BMI was 24.95 ± 3.87 kg/m². Post-treatment β-hCG levels were significantly higher in rupture cases: 10178.7 ± 11236.1 mIU/mL on day 4 and 9671.9 ± 10468.7 mIU/mL on day 7. ROC analysis yielded an AUC of 0.753 (cut-off: 2480.5 mIU/mL) on day 4 and 0.815 (cut-off: 1295.0 mIU/mL) on day 7, with sensitivities of 70% and specificities of 70% and 74%, respectively. NLR, MLR, PLR, and SII showed no significant predictive values. Hospitalization was longer in the surgical group (7.9 ± 5.8 days) compared to methotrexate-only cases (5.8 ± 3.9 days). β-hCG levels are reliable biomarkers for predicting rupture risk in ectopic pregnancies. Regular monitoring reduces surgical intervention and supports personalized treatment strategies.

## Introduction

Ectopic pregnancy is a pathology that occurs when fertilized ovum implants located outside the endometrium are considered a significant gynecological emergency^1^. Tubal ectopic pregnancy, particularly when located in the fallopian tubes, can lead to serious complications and poses a threat to maternal health if not diagnosed in the early stages^2^. Despite significant advancements in diagnostic methods and early intervention approaches, ectopic pregnancy remains a condition that requires attention because of its associated mortality and morbidity.

In the treatment of ectopic pregnancy, medical or surgical methods can be applied depending on factors such as the patient’s clinical condition and location of the pregnancy^3^. Methotrexate therapy is an effective medical option preferred in hemodynamically stable ectopic pregnancy cases that meet specific criteria and are diagnosed early^4^. Although this method reduces the need for surgical intervention, it requires careful monitoring of various laboratory and clinical parameters to predict treatment response^5^.

Among hemogram-based inflammatory indices, Neutrophil-to-Lymphocyte Ratio (NLR), Monocyte-to-Lymphocyte Ratio (MLR), Platelet-to-Lymphocyte Ratio (PLR), and Systemic Immune-Inflammation Index (SII) are widely used practical tools for assessing inflammatory processes^6,7^. These parameters have been shown to have prognostic value for various gynecological and obstetric conditions^8^. However, data on the prognostic role of these indices in ectopic pregnancy, particularly in cases treated with methotrexate, remain limited.

This study aimed to investigate the effects of hemogram-based inflammatory indices on treatment response and prognosis in ectopic pregnancy cases treated with methotrexate and to provide additional information to the literature on this topic.

## Materials and methods

This study was a retrospective investigation conducted at Kütahya Health Sciences University Evliya Çelebi Training and Research Hospital. Ethical approval for the study was obtained from the Non-Interventional Clinical Research Ethics Committee of Kütahya Health Sciences University (Approval No 2024/06-May 32 7, 2024). This study is retrospective in nature, and the requirement for informed consent was waived by the Non-Interventional Clinical Research Ethics Committee of Kütahya Health Sciences University in accordance with the relevant guidelines and regulations. This study was conducted in accordance with the principles outlined in the Declaration of Helsinki. All patient data were anonymized to ensure confidentiality, and strict measures were taken to protect the privacy and integrity of patient information throughout the study.

### Study population and patient selection

A total of 85 patients diagnosed with “ectopic pregnancy” and treated with methotrexate (MTX) between 2017 and 2024 at Kütahya Health Sciences University Evliya Çelebi Training and Research Hospital were included in this study. Ectopic pregnancy was diagnosed in accordance with the ACOG guidelines, based on the absence of an intrauterine pregnancy on transvaginal ultrasonography, the presence of a gestational sac or related structures in the adnexal region, and a positive pregnancy test. In cases where the diagnosis could not be confirmed and the pregnancy location was unknown, serial β-hCG measurements were performed and uterine curettage was used when necessary to assess the presence of chorionic villi. The diagnosis was confirmed on the basis of the β-hCG rise pattern. Clinically stable cases were closely monitored, and methotrexate was administered only to patients with a high suspicion or confirmed diagnosis of ectopic pregnancy. Patients diagnosed with non-tubal ectopic pregnancies (e.g., cervical or interstitial ectopic pregnancies), heterotopic pregnancies, or pregnancies of unknown location were excluded from the study.

Patients with the following characteristics were excluded from the study: known autoimmune diseases (e.g., rheumatoid arthritis) or the use of immunosuppressive drugs (e.g., steroids); acute or chronic liver or kidney failure; additional conditions that could affect inflammation and platelet-to-white blood cell counts, such as diabetes and/or cardiovascular diseases; pelvic inflammatory disease; history of major surgeries (e.g., extensive abdominal surgery) or active infections (e.g., coronavirus, influenza); smoking habits, cancer, or known hematological disorders; and conditions that contraindicate the use of methotrexate, including hypersensitivity to methotrexate, breastfeeding, psoriasis, active lung disease, or a history of peptic ulcers. Additionally, patients with embryonic cardiac activity, an initial β-hCG level > 5000 mIU/mL, an ectopic focus measuring > 4 cm on transvaginal ultrasonography, or those who refused blood transfusion were excluded from the study because of their potential to significantly impact prognosis and reduce the success of methotrexate treatment, as well as being defined as relative contraindications in the ACOG guidelines. The study population was defined based on these criteria, and the demographic characteristics of the patients (including age, body mass index (BMI), parity, and history of previous surgeries) as well as laboratory values were retrospectively obtained from the hospital database.

### Imaging and laboratory methods

After confirming the absence of intrauterine pregnancy using transvaginal ultrasonography (TVUSG), the evaluation included tubal ectopic pregnancy (TEP) masses, presence of an embryo (if any), and embryonic cardiac activity. The maximum diameter (and/or surface area) of the ectopic mass was measured using the same ultrasound system (GE Voluson 730 Expert System, General Electric Medical Systems, Milwaukee, WI, USA) equipped with a 5–9 MHz transvaginal probe, following standard protocols. The size of the ectopic focus was assessed using transvaginal ultrasonography by calculating the largest diameter and/or the surface area. In this study, owing to the limitations of retrospective data, only the surface area (mm^2^) was available for evaluation.

Blood samples collected during the initial admission of patients were placed into serum separator tubes (BD Vacutainer SST II Advance) for β-human chorionic gonadotropin (β-hCG) level analysis and into tubes containing anticoagulant (K2EDTA) (BD Vacutainer K2E) for complete blood count (CBC) analysis. Complete blood count measurements were performed using a hematology analyzer (Mindray BC-6800, Mindray Medical International Limited, Shenzhen, Guangdong, China), while β-hCG analyses were performed with an immunoassay analyzer (Roche Cobas e801, Roche Diagnostics International Limited, Rotkreuz, Switzerland) within approximately 30 min.

### Treatment protocol and follow-up

All patients were treated according to the current guidelines^9^ and protocols determined by obstetrics and gynecology specialists, considering their clinical stability. Hemodynamically stable patients with no contraindications to methotrexate (e.g., no more than a two-fold increase in liver enzymes, absence of acute abdominal symptoms) received a single-dose intramuscular injection of methotrexate at a dose of 50 mg/m². Treatment success was defined as a ≥ 15% decrease in β-hCG levels on days 4 and 7. Patients who failed to achieve this reduction or demonstrated an increase in β-hCG levels were reassessed, and a second dose of methotrexate (at the same dose) was planned. During follow-up, surgical management was initiated in cases with deterioration in vital signs (such as hypotension, tachycardia, or syncope), marked abdominal guarding and rebound tenderness on physical examination, increased free fluid on transvaginal ultrasonography, or suspicion of rupture. These decisions were made by a multidisciplinary team based on clinical monitoring data following a standardized treatment algorithm that prioritized patient safety.

### Inflammatory parameters and definitions

The following inflammatory indices were calculated using neutrophils (10^3^/µL), lymphocytes (10^3^/µL), monocytes (10^3^/µL), and platelets (10^3^/µL) obtained from the complete blood count results:

- **The neutrophil-to-lymphocyte Ratio (NLR) was** calculated by dividing the neutrophil count by the lymphocyte count.
- **Monocyte-to-Lymphocyte Ratio (MLR)**: Calculated by dividing monocyte count by lymphocyte count.
- **The platelet-to-lymphocyte Ratio (PLR) was** calculated by dividing the platelet count by the lymphocyte count.
- **The Systemic Immune-Inflammation Index (SII) was** calculated using the formula (neutrophil count × platelet count) / lymphocyte count^10^.

These values were obtained from complete blood count results during the initial admission. Additionally, for each case, β-hCG levels (at admission, on the day of MTX administration, and on days 4 and 7 post-MTX) and ultrasonographically measured sizes of the ectopic masses were recorded.

### Sample size and power analysis

The sample size calculation was performed using G*Power 3.1 software (Düsseldorf, Germany). The mean and standard deviation values reported by Dinç et al.^11^ were used as references. To compare the means of two independent groups, an effect size (d) of 0.891, an alpha error probability (α = 0.05), and a power (1–β) of 0.80 were considered. The calculations indicated that 42 patients, with at least 21 patients in each group, would be sufficient. The inclusion of 85 patients in our study was determined to have a sufficiently large sample size to obtain statistically significant results based on this power analysis.

### Statistical analysis

Data analysis was conducted using SPSS software (Version 27.0, Windows) released by IBM Corporation (Armonk, NY, USA). The normality of the data distribution was evaluated using the Kolmogorov-Smirnov test. Descriptive statistics were presented as mean, standard deviation, median, frequency, percentage, and minimum and maximum values. For variables following a normal distribution, the Independent Samples t-test was used to compare two independent groups, whereas the Mann-Whitney U test was applied for variables not following a normal distribution. The Pearson chi-square test was used to compare categorical variables. Statistical significance was set at *P* < 0.05. To determine the probability of rupture in ectopic pregnancy and to identify the optimal cutoff point for various β-hCG levels, a Receiver Operating Characteristic (ROC) analysis was performed. The results of ROC analysis, including the area under the curve (AUC), sensitivity, specificity, and identified threshold values, are presented.

## Results

The mean age of the participants was calculated as 32.1 ± 6.3 years, and the mean body mass index (BMI) was 24.95 ± 3.87 kg/m^2^. Among the hematological parameters, the mean white blood cell (WBC) count was 8.51 ± 2.22 × 10^3^/µL, the mean hemoglobin level was 12.6 ± 1.3 g/dL, the mean neutrophil count was 5.48 ± 2.05 × 10^3^/µL, and the mean platelet count was 283.1 ± 54.5 × 10^3^/µL (Table 1).

The mean initial β-hCG (Admission β-hCG) level of the participants was calculated as 2799.8 ± 3779.2 mIU/mL, while the mean β-hCG level on the day of methotrexate (MTX) administration was 3237.7 ± 4231.7 mIU/mL. The mean β-hCG level on day 4 post-MTX was 3721.0 ± 6013.8 mIU/mL, and on day 7 post-MTX, it was 3019.1 ± 5675.4 mIU/mL. The mean ectopic mass size was determined as 60.16 ± 223.04 mm^2^. Among the inflammatory indices, the mean neutrophil-to-lymphocyte ratio (NLR) was 2.78 ± 2.82, the mean monocyte-to-lymphocyte ratio (MLR) was 0.26 ± 0.26, the mean platelet-to-lymphocyte ratio (PLR) was 134.98 ± 62.91, and the mean systemic immune-inflammation index (SII) was 134.69 ± 69.41 (Table 2).

The mean age in the methotrexate group was 31.8 ± 6.0 years, while in the methotrexate with surgery group, it was 32.5 ± 7.6 years, with no statistically significant difference between the groups (*p* = 0.687). A significant difference was observed in β-hCG levels on day 4 post-MTX (2534.9 ± 3844.8 mIU/mL vs. 12369.6 ± 11597.6 mIU/mL; *p* = 0.003) and on day 7 post-MTX (1780.2 ± 3348.4 mIU/mL vs. 11899.3 ± 10605.3 mIU/mL; *p* < 0.001) between the two groups. No statistically significant differences were found between the groups in the other parameters (Table 3).

The mean time to methotrexate administration was 0.8 ± 1.4 days in the methotrexate group and 0.8 ± 1.6 days in the surgery group, with no statistically significant difference between the groups (*p* = 0.844). The total methotrexate dose was recorded as 103.4 ± 39.5 mg in the methotrexate group and 110.8 ± 46.5 mg in the surgery group, with no significant difference (*p* = 0.556). The total length of hospital stay was 5.8 ± 3.9 days in the methotrexate group and 7.9 ± 5.8 days in the surgery group, with a statistically significant difference between the groups (*p* = 0.049). The mean total red blood cell transfusion in the surgery group was recorded as 2.2 ± 1.1 units (Table 4).

In the non-rupture group, the mean initial β-hCG level was 2249.4 ± 3107.1 mIU/mL, whereas in the rupture group, it was 4636.9 ± 5432.3 mIU/mL, with a statistically significant difference (*p* = 0.061). A significant difference in β-hCG levels was observed between the non-rupture and rupture groups on the day of MTX administration (2502.3 ± 3388.0 mIU/mL vs. 5628.2 ± 5996.6 mIU/mL; *p* = 0.043). On day 4 post-MTX, the mean β-hCG level was 2569.9 ± 3894.5 mIU/mL in the non-rupture group and 10178.7 ± 11236.1 mIU/mL in the rupture group, with a statistically significant difference (*p* = 0.010). Similarly, on day 7 post-MTX, the β-hCG levels showed a significant difference between the groups (1811.1 ± 3393.4 mIU/mL vs. 9671.9 ± 10468.7 mIU/mL; *p* = 0.001). No statistically significant differences were found between the groups in terms of ectopic mass size (Ectopic Mass Size) or inflammatory parameters (NLR, MLR, and PLR) (Table 5).

**Fig. 1:**
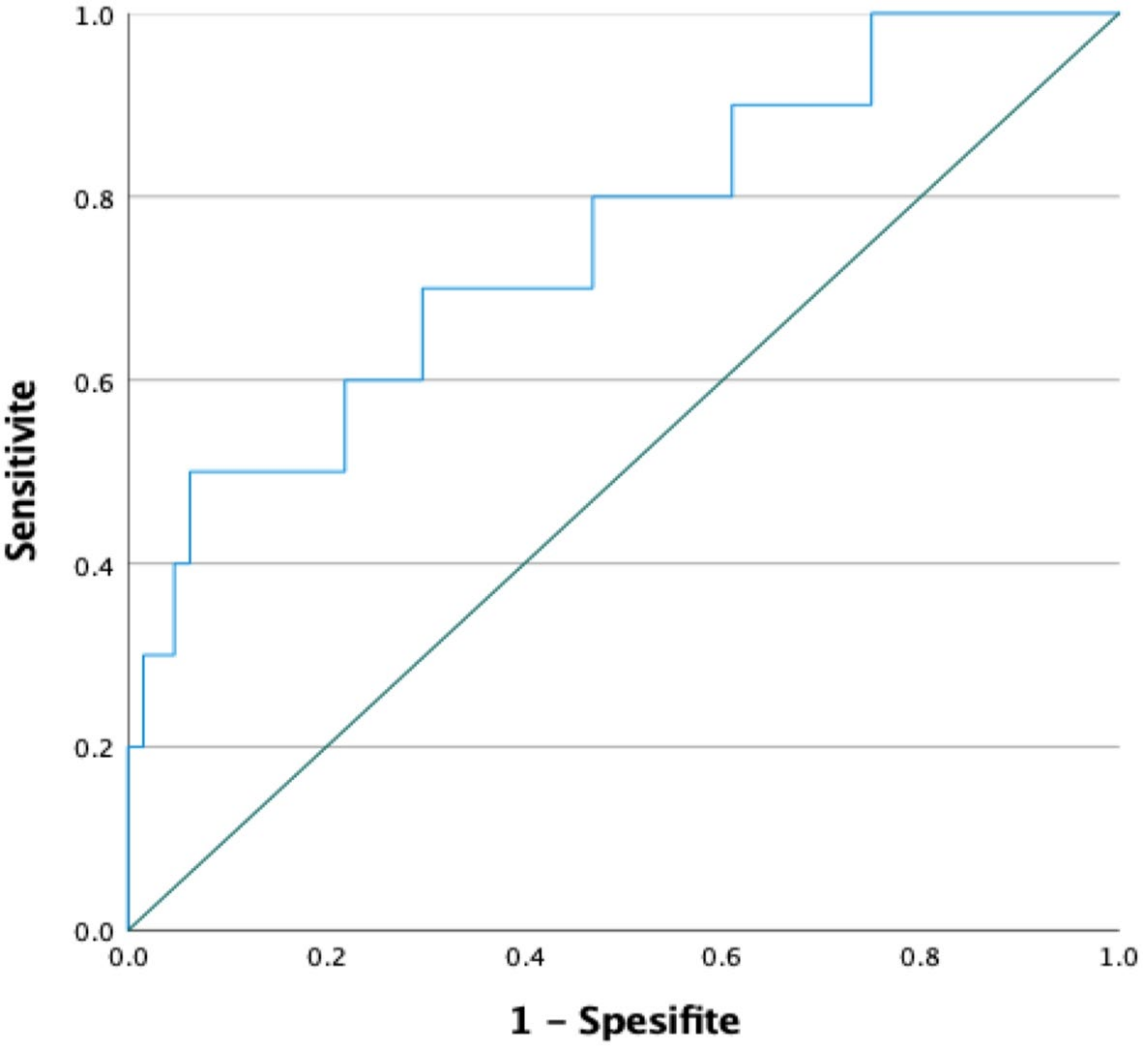
ROC curve for β-hCG level on day 4 of methotrexate treatment and ectopic pregnancy rupture.

The ROC curve for predicting the likelihood of ectopic pregnancy rupture based on β-hCG levels on day 4 of methotrexate treatment is presented in Fig. 1. The area under the curve (AUC) reflects the balance between sensitivity and specificity at a given cutoff value. This analysis demonstrated that β-hCG levels can serve as a potential biomarker for assessing rupture risk (Fig. 1).

In the ROC analysis for β-hCG levels on day 4 of methotrexate treatment, the area under the curve (AUC) was 0.753 (*p* = 0.010). The analysis determined the optimal cut-off value to be 2480.5 mIU/mL, with 70% sensitivity and 70% specificity. The confidence interval (95% CI) was calculated to be 0.582 for the lower bound and 0.925 for the upper bound (Table 6).

**Fig. 2:**
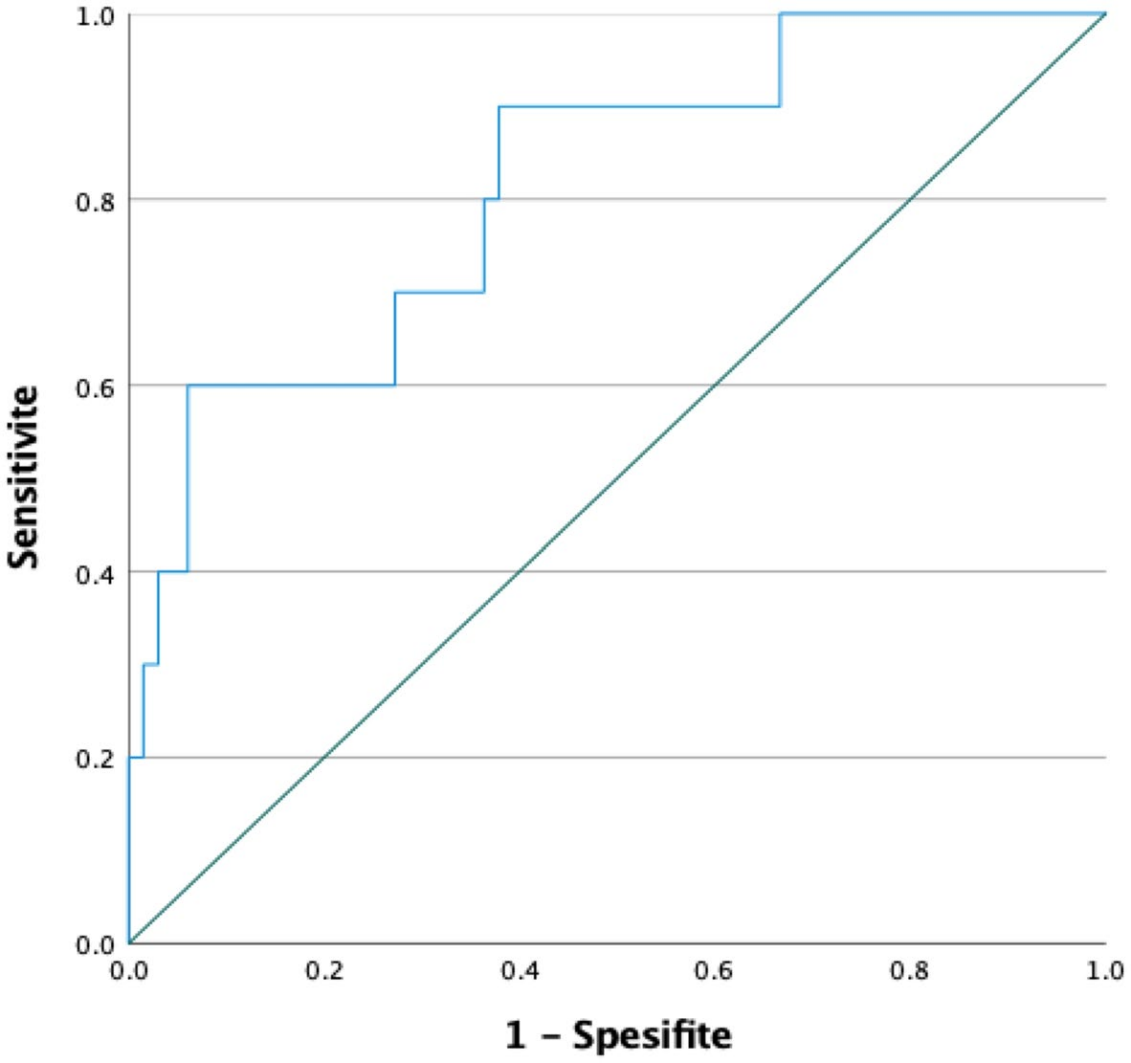
ROC Curve for β-hCG level on day 7 of methotrexate treatment and ectopic pregnancy rupture.

The ROC curve for predicting the likelihood of ectopic pregnancy rupture based on β-hCG levels on day 7 of methotrexate treatment is presented in Fig. 2. The curve (AUC) reflects the effectiveness of this biomarker in estimating rupture risk by considering the sensitivity and specificity values. The analysis results suggest that a specific cutoff value could have clinical significance in assessing rupture risk (Fig. 2).

In the ROC analysis for β-hCG levels on day 7 of methotrexate treatment, the area under the curve (AUC) was 0.815 (*p* = 0.001). The analysis determined the optimal cut-off value to be 1295.0 mIU/mL, with 70% sensitivity and 74% specificity. The confidence interval (95% CI) was calculated to be 0.672 for the lower bound and 0.958 for the upper bound (Table 7).

ROC analysis for predicting rupture based on inflammatory indices is also presented. AUC for NLR was 0.539 (*p* = 0.629), MLR it was 0.491 (*p* = 0.910), for the PLR it was 0.511 (*p* = 0.893), and for SII it was 0.580 (*p* = 0.323). None of the indices demonstrated a statistically significant predictive value for a rupture. The 95% confidence intervals for each parameter are also provided, ranging from the lower bound of 0.339 to the upper bound of 0.725 (Table 8).

## Discussion

In our study, β-hCG levels on days 4 and 7 of treatment were particularly significant in predicting the success of methotrexate treatment and the risk of rupture in ectopic pregnancy cases. This finding highlights the critical role of β-hCG monitoring in treatment planning and patient follow-up. On the other hand, the lack of a significant difference in inflammatory parameters, such as neutrophil-to-lymphocyte ratio and monocyte-to-lymphocyte ratio, in relation to rupture risk or the need for additional surgery suggests that these markers alone may not be decisive in clinical decision-making. Additionally, hospital stays were shorter in cases managed solely with methotrexate, whereas prolonged hospitalization was required in patients needing surgical intervention due to additional procedures and the need for close monitoring. These findings indicate that early and appropriate patient selection combined with regular β-hCG monitoring could be a key strategy in improving methotrexate treatment success and reducing the need for surgical intervention. In line with this, a study by Fıratlıgil et al. examined a large cohort of ectopic pregnancy cases requiring life-saving urgent surgery and reported that 93 of the 469 patients (19.8%) underwent surgical intervention following failed methotrexate treatment^12^. These patients demonstrated significant differences in clinical and laboratory parameters compared with those who underwent primary surgical treatment, including lower rates of intra-abdominal free fluid and smaller ectopic mass diameters. The authors emphasized that early diagnosis, appropriate patient selection, and close monitoring were key to reducing the morbidity associated with rupture and emergency surgery. These findings support the clinical importance of β-hCG follow-up and align with our results regarding the predictive value of β-hCG on days 4 and 7 post-treatment.

In a study by Dereli et al., hemogram-based inflammatory parameters such as NLR, PLR, MLR, and SII were reported to be statistically significant in predicting methotrexate success, with these parameters being lower in the group with a reduced need for surgery. In contrast, our study did not detect significant differences in these parameters (NLR, PLR, MLR, and SII) between groups^13^. Therefore, the results of Dereli et al. and our findings do not fully align, particularly regarding the prognostic importance of the inflammatory indices. Dereli et al. observed no statistically significant difference when comparing ROC curves; however, the AUC value for neutrophils was higher than that for SII in predicting methotrexate success. Nevertheless, relying solely on a single cell line for clinical decision making may not be appropriate. Methotrexate response may vary in patients with similar neutrophil and lymphocyte counts or comparable NLRs when differences in platelet counts are considered. This variability is supported by studies comparing patients with intact or ruptured tubal ectopic pregnancies, which have reported differing platelet counts^14–16^.

In their study on leukocyte levels in patients with ectopic pregnancy (EP), Turgut et al. found that leukocyte levels were significantly higher in the EP group without tubal rupture than in the control group^17^. However, no statistically significant difference was observed in the mean platelet volume (MPV) between cases with and without rupture. Our findings align with those of previous studies suggesting that elevated serum β-hCG levels may correlate with the number of trophoblasts and depth of tubal invasion^18–20^. For instance, Darkhaneh et al.^21^ proposed a threshold of 1750 IU/mL for predicting tubal rupture, whereas Elito Jr. et al.^22^ associated a level of 2906 mIU/mL with advanced (stage III) invasion. In our study, the early post-treatment cut-off value for β-hCG was determined to be 2480.5 mIU/mL on day 4 of MTX treatment and 1295.0 mIU/mL on day 7, with significant sensitivity and specificity observed in both measurements. These results are consistent with existing literature, which suggests that β-hCG levels above specific thresholds may increase the risk of tubal damage or rupture. Therefore, close monitoring of β-hCG levels, particularly before treatment and on days 4 and 7 post-MTX administration, may serve as a clinically significant guide for predicting and potentially preventing early surgical intervention in the management of ectopic pregnancy.

The findings of this study emphasize the importance of β-hCG levels in predicting rupture in ectopic pregnancy cases treated with methotrexate. Specifically, β-hCG levels on days 4 and 7 post-treatment demonstrated statistically significant differences between the rupture and non-rupture groups, suggesting their potential as reliable biomarkers for clinical decision-making. ROC analyses revealed an AUC of 0.753 for β-hCG on day 4 and 0.815 on day 7, indicating a strong predictive value. However, inflammatory indices, such as NLR, MLR, PLR, and SII, were found to lack statistically significant predictive values for rupture (Table 8). This suggests that the inflammatory response in ectopic pregnancy may depend not only on systemic inflammation markers, but also on multifactorial interactions, including localized inflammation, tissue damage, and other clinical parameters. These findings reaffirm the critical role of β-hCG levels in clinical risk assessment and individualized treatment strategies while highlighting the need for further studies to identify more effective inflammatory markers for predicting rupture.

This study has several strengths. This is one of the few studies that directly compares hemogram-based inflammatory indices with serial β-hCG measurements in predicting methotrexate treatment outcomes and rupture risk in ectopic pregnancy. The identification of specific β-hCG cutoff values on days 4 and 7 post-treatment provides clinically actionable information that may assist physicians in early risk stratification and decision-making. Additionally, the exclusion of patients with confounding inflammatory or systemic conditions enhances the internal validity of our findings. However, this study has several limitations. There was a notable imbalance in the number of patients in the rupture and non-rupture groups. In particular, the small number of patients in the rupture group may have limited the statistical power of the comparisons. Additionally, the single-center retrospective design, along with a lack of geographic and demographic diversity, may restrict the generalizability of the findings. Furthermore, while inflammatory indices, such as NLR, MLR, PLR, and SII, were assessed, more comprehensive inflammatory or biochemical markers were not included. Finally, although β-hCG kinetics were analyzed in detail, other clinical variables such as pain severity or ultrasound features were not systematically evaluated. These limitations suggest that our findings should be interpreted cautiously and validated in future large-scale prospective multicenter studies.

In conclusion, this study highlights that although hemogram-based inflammatory indices such as NLR, PLR, MLR, and SII may offer limited information, they are not reliable predictors of methotrexate success or rupture risk in ectopic pregnancy. In contrast, serial β-hCG measurements, particularly on days 4 and 7 after treatment, proved to be significant indicators of both treatment efficacy and potential for rupture. These findings underscore the importance of regular biochemical monitoring of static inflammatory parameters in clinical decision making. This study contributes to the current literature by challenging the prognostic dominance of inflammatory indices and reaffirming β-hCG kinetics as a central tool for risk stratification. Future prospective studies with larger and more diverse populations are necessary to validate these findings and identify novel biomarkers that may enhance individualized treatment approaches in ectopic pregnancy management.

**Table 1:** Demographic characteristics of participants.

	Mean ± S.D./Count (%)
Age (years)	32.1 ± 6.3
BMI (kg/m^2^)	24.95 ± 3.87
WBC (10^3^/µL)	8.51 ± 2.22
Hemoglobin (g/dL)	12.6 ± 1.3
Hematocrit (%)	37.9 ± 3.5
Lymphocyte (10^3^/µL)	2.34 ± 0.74
Monocyte (10^3^/µL)	0.60 ± 0.78
Neutrophil (10^3^/µL)	5.48 ± 2.05
Platelet (10^3^/µL)	283.1 ± 54.5

Data are presented as mean ± standard deviation and number (percentage). Abbreviations: BMI: Body Mass Index.

**Table 2:** Hematological parameters of participants.

	Mean ± S.D.
Admission β-hCG (mIU/mL)	2799.8 ± 3779.2
MTX day β-hCG (mIU/mL)	3237.7 ± 4231.7
MTX day 4 β-hCG (mIU/mL)	3721.0 ± 6013.8
MTX day 7 β-hCG (mIU/mL)	3019.1 ± 5675.4
Ectopic Mass Size (mm²)	60.16 ± 223.04
NLR	2.78 ± 2.82
MLR	0.26 ± 0.26
PLR	134.98 ± 62.91
SII	134.69 ± 69.41

**Table 3:** Comparison of demographic and hematological parameters between methotrexate-only and methotrexate with surgery groups.

	Metotrexate (*n* = 71)	Mtx and surgery (*n* = 14)	p value
	Mean ± S.D.	Mean ± S.D.	
Age (years)	31.8 ± 6.0	32.5 ± 7.6	0.687 ^b^
BMI (kg/m^2^)	25.07 ± 3.97	24.03 ± 3.5	0.362 ^a^
Parity	0.8 ± 0.9	0.9 ± 0.9	0.441 ^b^
WBC (10^3^/µL)	8.39 ± 2.18	8.82 ± 2.6	0.516 ^a^
Hemoglobin (g/dL)	12.5 ± 1.3	12.6 ± 1.4	0.948 ^b^
Hematocrit (%)	37.9 ± 3.6	37.6 ± 3.5	0.813 ^a^
Lymphocyte (10^3^/µL)	2.35 ± 0.73	2.36 ± 0.8	0.951 ^a^
Monocyte (10^3^/µL)	0.62 ± 0.9	0.50 ± 0.2	0.619 ^b^
Neutrophil (10^3^/µL)	5.37 ± 1.95	5.76 ± 2.5	0.803 ^b^
Platelet (10^3^/µL)	281.9 ± 57.3	290.9 ± 39.4	0.580 ^a^
Admission β-hCG	2301.5 ± 3140.4	4721.1 ± 5647.8	0.098 ^b^
MTX day β-hCG	2563.0 ± 3437.2	5787.1 ± 6216.2	0.054 ^b^
MTX day 4 β-hCG	2534.9 ± 3844.8	12369.6 ± 11597.6	**0.003** ^b^
MTX day 7 β-hCG	1780.2 ± 3348.4	11899.3 ± 10605.3	**< 0.001** ^b^
NLR	2.70 ± 2.8	3.03 ± 3.2	0.813 ^b^
MLR	0.26 ± 0.28	0.24 ± 0.18	0.740 ^b^
PLR	134.00 ± 63.8	140.63 ± 67.3	0.804 ^b^
SII	136.43 ± 71.9	131.52 ± 52.3	0.813 ^b^

Data are presented as mean ± standard deviation.

BMI: Body Mass Index, WBC: White Blood Cell Count, NLR: Neutrophil-to-Lymphocyte Ratio, MLR: Monocyte-to-Lymphocyte Ratio, PLR: Platelet-to-Lymphocyte Ratio, SII: Systemic Immune-Inflammation Index, β-hCG: Beta-Human Chorionic Gonadotropin.

^a^Independent Samples t-test, ^b^Mann-Whitney U test. Statistically significant p-values are highlighted in bold.

**Table 4:** Comparison of treatment and hospitalization durations between methotrexate-only and methotrexate with surgery groups.

	Metotrexate (*n* = 71)	Mtx and surgery (*n* = 14)	*p* value
	Mean ± S.D.	Mean ± S.D.	
Days after admission until MTX	0.8 ± 1.4	0.8 ± 1.6	0.844 ^b^
Total methotrexate dose	103.4 ± 39.5	110.8 ± 46.5	0.556 ^b^
Total length of hospital stay (days)	5.8 ± 3.9	7.9 ± 5.8	**0.049** ^**a**^
Total red blood cell transfusion	-	2.2 ± 1.1	-

Data are presented as mean ± standard deviation.

Mtx: Methotrexate.

^a^Independent Samples t-test, ^b^Mann-Whitney U test. Statistically significant p-values are highlighted in bold.

**Table 5:** Comparison of inflammatory parameters between rupture and non-rupture groups.

	No rupture (*n* = 69)	Rupture (*n* = 16)	p value
	Mean ± S.D.	Mean ± S.D.	
Admission β-hCG (mIU/mL)	2249.4 ± 3107.1	4636.9 ± 5432.3	0.061 ^b^
MTX day β-hCG (mIU/mL)	2502.3 ± 3388.0	5628.2 ± 5996.6	**0.043** ^**b**^
MTX day 4 β-hCG (mIU/mL)	2569.9 ± 3894.5	10178.7 ± 11236.1	**0.010** ^**b**^
MTX day 7 β-hCG (mIU/mL)	1811.1 ± 3393.4	9671.9 ± 10468.7	**0.001** ^**b**^
Ectopic mass size (^mm2^)	2.70 ± 2.84	2.98 ± 2.96	0.629 ^b^
NLR	0.26 ± 0.3	0.24 ± 0.17	0.910 ^b^
MLR	134.40 ± 64.6	138.08 ± 63.0	0.893 ^b^
PLR	136.35 ± 72.7	132.49 ± 50.2	0.744 ^b^

Data are presented as mean ± standard deviation. Mtx: Methotrexate, NLR: Neutrophil-to-Lymphocyte Ratio, MLR: Monocyte-to-Lymphocyte Ratio, PLR: Platelet-to-Lymphocyte Ratio, SII: Systemic Immune-Inflammation Index. ^a^Independent Samples t-test, ^b^Mann-Whitney U test. Statistically significant p-values are highlighted in bold.

**Table 6:** ROC analysis for β-hCG level on day 4 of methotrexate treatment and ectopic pregnancy rupture.

AUC	*p* value	Sensitivity	Specifity	Cut-off	Asymp. 95% Conf. Int.	
					Lower Bound	Upper Bound
0.753	**0.010**	0.70	0.70	2480.5	0.582	0.925

Significant values are in [bold].

**Table 7:** ROC analysis for β-hCG level on day 7 of methotrexate treatment and ectopic pregnancy rupture.

AUC	*p* value	Sensitivity	Specifity	Cut-off	Asymp. 95% Conf. Int.	
					Lower bound	Upper bound
0.815	**0.001**	0.70	0.74	1295.0	0.672	0.958

Significant values are in [bold].

**Table 8:** ROC analysis for predicting rupture based on inflammatory indices.

	AUC	Std. Error	*p* value	Asymp. 95% Conf. Int.	
				Lower bound	Upper bound
NLO	0.539	0.075	0.629	0.392	0.686
MLO	0.491	0.078	0.910	0.339	0.643
PLO	0.511	0.079	0.893	0.355	0.667
SII	0.580	0.074	0.323	0.434	0.725

## Data Availability

The data that support the findings of this study are available from Kütahya Health Sciences University. However, restrictions apply to the availability of these data, which were used under license for the current study and are not publicly available. Data are, however, available from the corresponding author upon reasonable request and with permission from Kütahya Health Sciences University.
